# GTP-binding protein Era: a novel gene target for biofuel production

**DOI:** 10.1186/s12896-015-0132-1

**Published:** 2015-03-24

**Authors:** Gerben P Voshol, Vera Meyer, Cees A M J J van den Hondel

**Affiliations:** Molecular Microbiology and Biotechnology, Institute of Biology Leiden, Leiden University, Leiden, The Netherlands; International Research Centre “Biotechnologies of the Third Millennium”, ITMO University, Saint-Petersburg, Russia; Applied and Molecular Microbiology, Institute of Biotechnology, Berlin University of Technology, Berlin, Germany

**Keywords:** Biofuels, Cyanobacteria, GTP-binding protein Era, Fatty acids, Hydrocarbons, *Synechococcus elongatus* PCC 7942

## Abstract

**Background:**

Biodiesel production using cyanobacteria is a promising alternative to fossil fuels. In this study we created a transposon library of *Synechococcus elongatus* PCC 7942 in order to identify novel gene targets for enhanced fatty acid and hydrocarbon production. The transposon library was subsequently screened for desirable traits using macro- and microscopic observations as well as staining with the lipophilic dye Nile Red.

**Results:**

Based on the screening results, we selected a single mutant, which has an insertion in the gene encoding for the GTP-binding protein Era. We subsequently verified the phenotype-genotype relation by overexpression, reintroducing and complementing the mutation. Overexpression of *era* caused a reduction in the cell size in the late exponential phase of growth and an increase in the total amount of intracellular fatty acids. Reintroduction of the inactivated transposon caused a significant increase in the cellular length as well as changes in the amounts of individual hydrocarbons and fatty acids. Ectopic complementation of this mutation fully restored the hydrocarbon production profile to that of wild-type and partially restored the fatty acid production. Moreover, the cellular size was significantly smaller than that of the inactivated transposon mutant.

**Conclusions:**

The GTP-binding protein Era has never been studied in cyanobacteria and proved to be an essential gene for *S. elongatus* PCC 7942. We also found that this protein is important for hydrocarbon and fatty acid metabolism as well as determination of the cell size in PCC 7942. Our results suggest that the GTP-binding protein Era can be used as a novel target for further improvement of biofuel precursors production.

**Electronic supplementary material:**

The online version of this article (doi:10.1186/s12896-015-0132-1) contains supplementary material, which is available to authorized users.

## Background

Biodiesel produced by photosynthetic microorganisms, such as eukaryotic algae and cyanobacteria, provides a promising alternative to reduce our reliance on fossil fuels. Biodiesel can be produced either directly by these organisms or from their biomass. This fuel is carbon neutral, renewable and its use requires minimal changes in the current structure of fuel delivery and consumption. However, the relatively low productivity and the high cost of harvesting the biomass present mayor limitations for commercialization of cyanobacteria-derived biodiesels [1,2].

These limitations have been mainly addressed by using a rational approach [3-5]. The rational approach involves designing strains with enhanced biodiesel precursor production (e.g. fatty acids, hydrocarbons) by modifying and/or introducing known metabolic pathways. The most common modifications include the introduction of a heterologous thioesterase and removal of the endogenous acyl-ACP synthetase. The introduced thioesterase is capable of hydrolysing the acyl-ACP molecule and thus releases the fatty acid, the major precursor of biodiesel [6]. The acyl-ACP synthetase can reactivate free fatty acids by attaching them to the ACP-molecule [7]. By introducing a thioesterase in combination with disrupting the endogenous acyl-ACP synthetase, one can significantly enhance fatty acid production and secretion in cyanobacteria [1,4,5].

However, despite some success, the reported fatty acid yields are still not sufficient for large-scale production. This is partly due to the fact that the bulk of the fatty acids remain within the cell. Changing the cell structure in such a way that the cells can auto-flocculate or elongate might help to harvest the remaining biomass more efficiently [8]. Since the rational approach is guided by existing knowledge about the cellular processes involved in fatty acid biosynthesis and/or transport, it can limit strain improvement due to missing information on key intermediates, regulators, competing pathways etc. Therefore, combining the rational approach with random approaches (e.g. transposon mutagenesis) may result in the identification of novel genes involved in efficient biodiesel precursor production.

Random mutagenesis has been successfully used in cyanobacteria to isolate filamentous [9] and grazing resistant mutants [10] as well as to identify environmentally responsive genes [11] and genes involved in polyhydroxybutyrate synthesis [12]. Furthermore, this approach was previously applied to isolate genes involved in fatty acid production in *Escherichia coli* [13]. However, genes identified by Hoover and colleagues (2012) in *E. coli* lack obvious orthologous genes in cyanobacteria. Moreover, to our knowledge, random mutagenesis has never been applied to isolate lipid-overproducing mutants in cyanobacteria.

In this study we used *Synechococcus elongatus* PCC 7942 which is amenable to genetic modification and has a fully sequenced genome. This strain produces the major biodiesel precursors and lacks the ability to synthesize PHB (a competing pathway for biofuel production) [12]. These properties make *S. elongatus* PCC 7942 a good model strain to isolate mutants with an enhanced fatty acid production. The construction and analysis of a transposon generated mutant library, resulted in the identification of a gene that directly or indirectly affects fatty acid and hydrocarbon production. Moreover, this gene, named *era*, is also involved in determining the cellular morphology of PCC 7942. To our knowledge this gene has never been analysed in cyanobacteria.

## Results

### Isolation of a putative fatty acid overproducing mutant by random mutagenesis

To obtain a mutant library, we haven chosen to construct a transposon library using plasmid pRL1063a, which contains a Tn5 transposon with a kanamycin resistance marker, promoterless *luxAB* reporter genes and an origin of replication which functions in *E. coli*, but not in PCC 7942. This library, contained approximately 600 individual mutants, was subsequently screened for mutants with beneficial phenotypes such as filamentous and auto-flocculating growth, higher lipid content and phenotypic stability. Out of the initial 600 mutants a single strain was selected for further study based on its (i) elongated cell morphology, (ii) enhanced fluorescence after staining with the lipophilic dye Nile Red and (iii) phenotypic stability.

**Figure 1:**
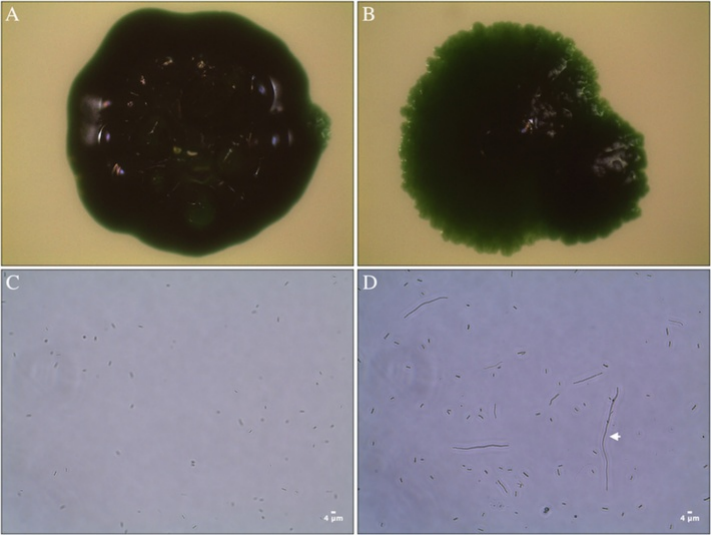
**Macro (A and B) and microscopic morphology (C and D) of wild-type PCC 7942 (A and C) and mutant 2A01 (B and D).** Macroscopic morphology was visualized after growth on plate using a stereomicroscope. Microscopic morphology was visualized by phase-contrast microscopy. Scale bars represent 4 μm. Example of an elongated cell is indicated with an arrow (97 μm long).

The selected mutant, 2A01, was both phenotypically and genotypically stable for a period of at least 3 months (verified by liquid to liquid sub-cultivation and PCR). This mutant showed a different colony morphology compared to PCC 7942 (Figure 1A and B). 2A01 has a round, slightly elevated colony morphology with irregular shaped edges on plate (Figure 1B) while PCC 7942 is round, raised with an entire smooth edge (Figure 1A). Phase contrast microscopy revealed that cultures of mutant 2A01 contained a heterogeneous mix of short cells similar in size to PCC 7942 and long elongated cells some more than 30 times the length of PCC 7942 (Figure 1C and D).

**Figure 2:**
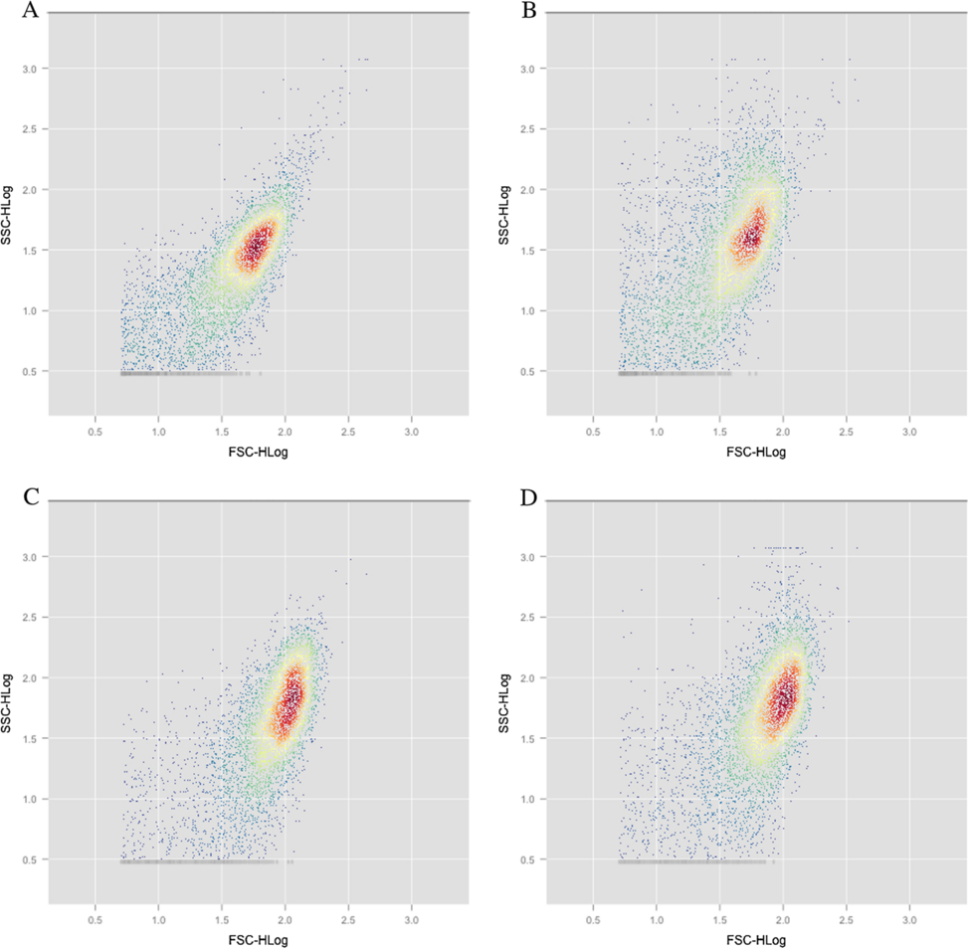
**Side scatter (cellular complexity) versus front scatter (cell length) as determined using flow cytometry.** PCC 7942 **(A and B)** and mutant 2A01 **(C and D)** grown in liquid media for 48 hours either unstained **(A and C)** or stained **(B and D)** with the lipophilic dye Nile red.

We also used flow cytometry to determine cellular morphology (size and complexity) of PCC 7942 and 2A01 stained with Nile Red (Figure 2B and D) or unstained (Figure 2A and A). Stained cells are very similar to unstained cells in respects to their cellular size (forward scatter), however the complexity (side scatter) after staining with Nile Red is slightly, but significantly higher. Comparison of cells of PCC 7942 (Figure 2A) and 2A01 (Figure 2C), furthermore indicates that cells of 2A01 are both larger in overall size and complexity than PCC 7942.

**Figure 3:**
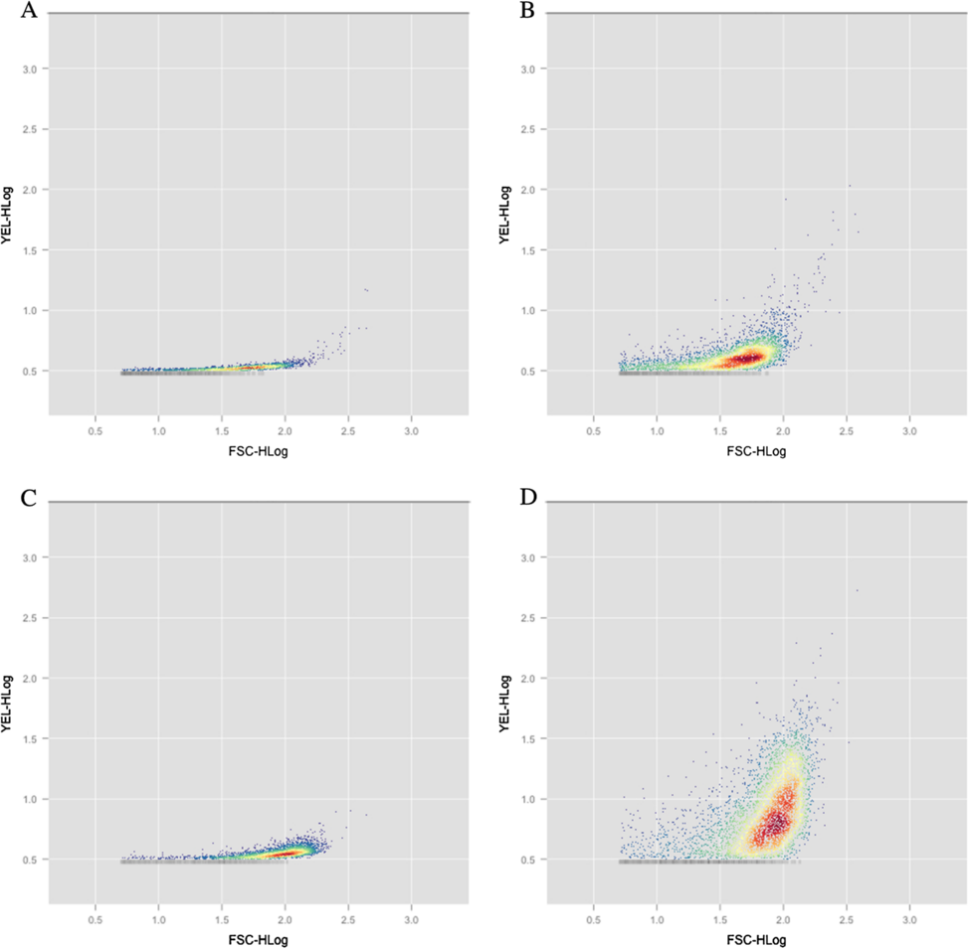
**Yellow fluorescence versus front scatter (cell length) as determined using flow cytometry.** PCC 7942 **(A and B)** and mutant 2A01 **(C and D)** grown in liquid media for 48 hours either unstained **(A and C)** or stained **(B and D)** with the lipophilic dye Nile red.

The largest differences between PCC 7942 and 2A01 where observed when comparing the yellow fluorescence (indication of lipid content) of Nile Red stained cells. In general, unstained cells showed a low amount of yellow fluorescence compared to both red and green fluorescence (Additional file 1: Figure S1 and S2). Cells stained with Nile Red showed an increase of yellow fluorescence (Figure 3A and C versus B and D) and 2A01 showed a higher increase in yellow fluorescence even at comparable cellular size (forward scatter) than PCC 7942.

### Transposon insertion site

To identify the insertion site of the transposon, genomic DNA was isolated, digested, re-ligated and transformed into *E. coli* DH5α. Plasmid DNA was extracted from colonies that acquired antibiotic resistance and restriction patterns were compared to identify additional transposition events. Based on their distinct restriction patterns, DNA of two isolated plasmids was sequenced. Both sequences showed the insertion of the transposon into the 3 prime region of gene Synpcc7942_0160 annotated as a GTP-binding protein Era. The insertion caused the formation of an early stop codon leading to a truncated protein lacking the last 20 amino acids. The GTP-binding protein era of PCC 7942 contains both a GTP binding and ATP hydrolysing domain at its N terminus and a type 2 K homology (KH) domain at its C-terminus (Additional file 1: Figure S3). In PCC 7942, this gene is the first gene in an operon containing a total of 8 genes [14].

### Verification genotype-phenotype relationship

To identify the genotype, phenotype relationship of mutant 2A01 and to elucidate the function of the GTP-binding protein Era in PCC 7942, several rational approaches were used.

To test the effect of overexpression of the GTP-binding protein Era in PCC7942, a construct containing an IPTG inducible promoter followed by a ribosomal binding site and the full-length wild type era gene was made (pNS3:era). Insertion of this construct into the neutral site 3 from PCC 7942 [15] was obtained after transformation, resulting in strain Se:era. Full segregation was verified by PCR amplification. As a negative control, a similar construct lacking the ribosomal binding site and era gene (pNS3) was inserted into the neutral site 3 from PCC 7942 (PCC 7942).

To reconstruct the original phenotype, the plasmid containing the transposon flanked by genomic DNA isolated from 2A01 was reinserted into the genome of PCC 7942 after inactivating the transposase by introducing a frame shift mutation. This mutant showed the correct insertion and was fully segregated (as shown by PCR amplification). This mutant was complemented by introducing the overexpression construct pNS3:era, resulting in strain se:∆era + era and as a negative control the construct lacking the ribosomal binding site and era gene was inserted into neutral site 3, mutant se:∆era.

In addition, a knockout construct was made in which the coding region of the era gene was replaced by a Km resistance cassette. Mutants obtained revealed an elongated phenotype (Additional file 1: Figure S4). However after subcultivation, these initial mutants either lost viability, or the wild type phenotype was restored.

### Growth

**Figure 4:**
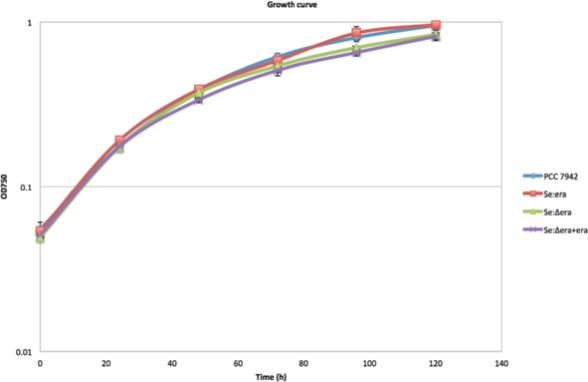
**Cellular growth of wild type PCC 7942, strain Se:era, Se:∆era and Se:∆era + era.** Growth was measured at 24 hour intervals using the optical density at 750 nm. Error bars represent the standard deviation of four replicates.

All strains show similar specific growth rates as measured using OD750 (Figure 4). The growth rates of mutant strains were 0.041 ± 0.002, 0.042 ± 0.001 and 0.039 ± 0.001 h^−1^ for Se:era, Se:Δera and Se:Δera + era respectively. These specific growth rates were not significantly different from that of PCC 7942 (0.041 ± 0.002 h^−1^). The final biomass of mutant strains at time of harvesting was also not significantly different compared to PCC 7942.

### Cellular morphology: length distribution during growth

**Figure 5:**
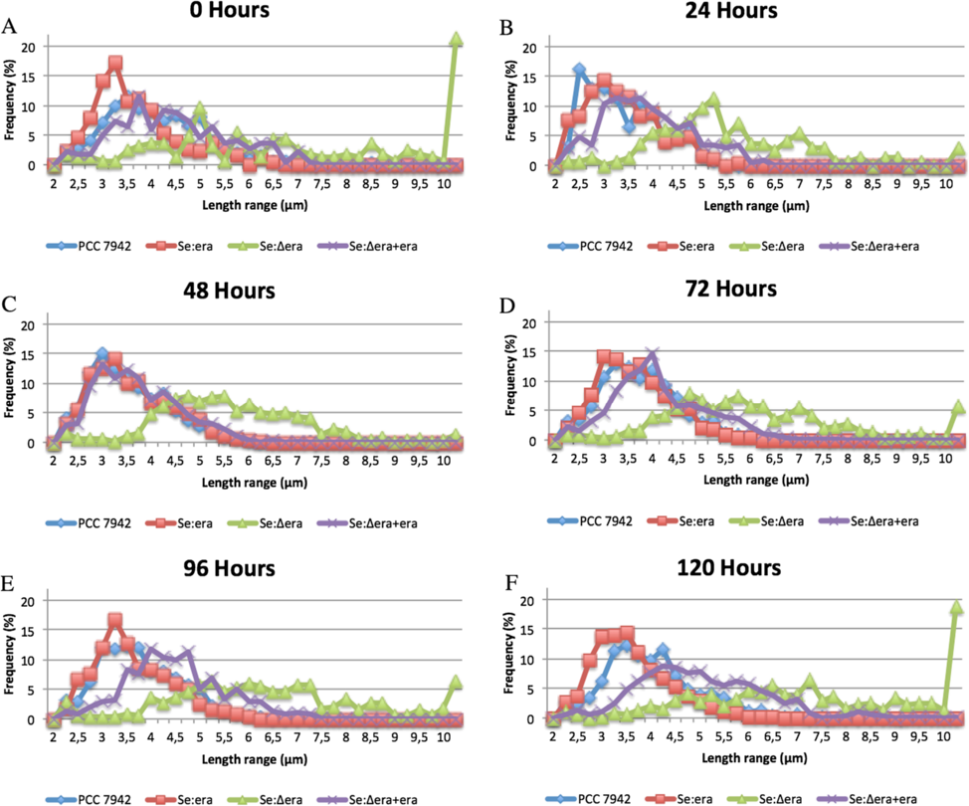
**Distribution of cell lengths in liquid cultures of PCC 7942 and mutant Se:era, Se:∆era and Se:∆era + era after 0 (A), 24 (B), 48 (C), 72 (D), 96 (E) and 120 (F) hours.** Lengths of cells of each strain was determined at 24 hour intervals by taking pictures using a phase contrast microscope and using imageJ to determine their cell size.

To monitor changes in cell size, aliquots from cultures were examined using a phase contrast microscopy (Figure 5).

Strain se:era, containing an additional copy of era, is significantly smaller at the start of the experiment (at 0 hours) compared to WT with an median of 3.3 and 3.8 μm, respectively. At time points 24, 48 (exponential phase) and 72 hours (early stationary phase) both PCC 7942 and se:era have a similar median cell length of 3.1, 3.3 and 3.4-3.5 μm (se:era-PCC 7942), respectively. At 96 hours, the median cell length of PCC 7942 is significantly larger compared to se:era (3.5 μm versus 3.3 μm). At the time of harvesting (120 hours) the cell length of PCC 7942 elongated further to 3.8 μm while cells from se:era had a median cell length of 3.4 μm.

Strain se:∆era has a significantly larger median cell size compared to PCC 7942 at all time points. Se:∆era had a median cell length of 6.4 μm at the time of inoculation (0 hours). This cell length decreased in the first 24 hours of growth to 5.2 μm. After this time point the cell length increase to 5.3, 5.6, 6.0 and 7.0 μm at 48, 72, 96 and 120 hours, respectively.

Strain se:∆era + era has a significantly reduced cell length compared to se:∆era, but is larger than PCC 7942 at all time points (Figure 5).

### Cellular morphology: microscopy

**Figure 6:**
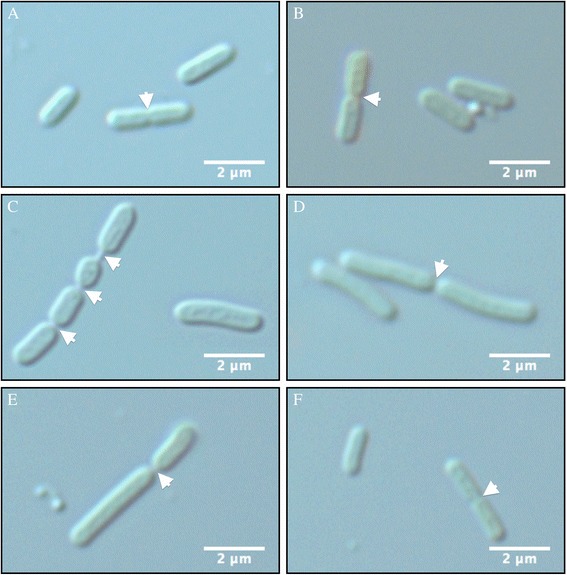
**Morphology of PCC 7942 (A), mutant Se:era (B), Se:∆era (C, D and E) and Se:∆era + era (F) grown in liquid media for 48 hours.** Scale bars represent 2 μm. The site of septa formation are indicated with arrows.

Differential interference microscopic analysis of the mutant and wild-type strains revealed that strain PCC 7942, se:era and se:∆era:era appear very similar to each other in cell size at 48 hours (Figure 6A, B and F). This result is similar to the results obtained by measuring the cell size (Figure 5C). Furthermore, septum formation occurs in a symmetric manner for all these mutants.

Mutant se:∆era however has both elongated cells and cell similar in size to PCC 7942. Constrictions can occur similar to PCC 7942, at the middle of the cell (Figure 6D). However, constrictions are often observed more towards the cellular pole (Figure 6E) or sometimes multiple times within a single cell (Figure 6C). Furthermore, full separation of the daughter cells does also not always occur leading to the formation of cells arrested at the pre-divisional two-cell stage.

### Fatty acid and hydrocarbon profile PCC 7942

**Figure 7:**
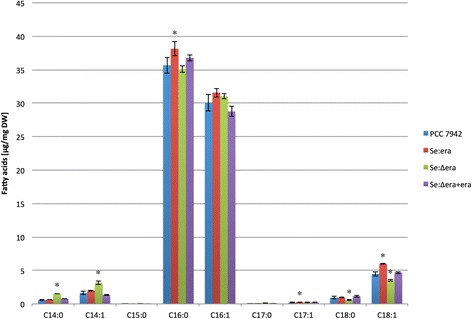
**Quantitative measurement of intracellular fatty acids in PCC 7942, strain Se:era, Se:∆era and Se:∆era + era.** Metabolites indicated with an asterisk * are significantly different (p < 0.05) from those of strain PCC 7942. Error bars represent the standard deviation of four replicates.

PCC 7942 contains a mixture of saturated and unsaturated fatty acids ranging from C14 to C18. Palmitic (C16:0) and palmitoleic acid (C16:1) represent the main intracellular fatty acids of PCC 7942 (Figure 7 and Additional file 2: Table S1). Together these fatty acids represent approximately 89% (35.7 ± 1.2 μg C16:0/mg DW and 30.1 ± 1.2 μg C16:1/mg DW) of the total fatty acids (73.6 ± 2.7 μg/mg DW). The remainder mainly consists of saturated and unsaturated C14 and C18 fatty acids followed by trace amounts of odd chain C15:0, C17:0 and C17:1.

**Figure 8:**
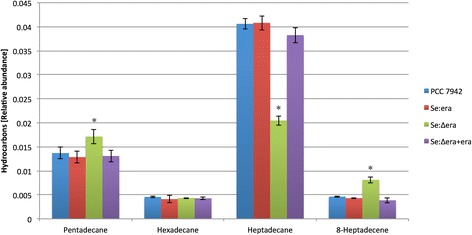
**Quantitative measurement of intracellular hydrocarbons in PCC 7942, strain Se:era, Se:∆era and Se:∆era + era.** Metabolites indicated with an asterisk * are significantly different (p < 0.05) from those of strain PCC 7942. Error bars represent the standard deviation of four replicates.

PCC 7942 is able to synthesize both alkanes and alkenes (Figure 8 and Additional file 2: Table S1). The alkanes synthesized by PCC 7942 are mostly heptadecane (C17:0), followed by pentadecane (C15:0) and hexadecane (C16:0). The only alkene that we detected was 8-heptadecene at levels comparable to those of hexadecane.

### Fatty acid and hydrocarbon profile Se:era

Strain Se:era, which contains an additional era gene inserted at neutral site 3, has a significant increase in the total amount of fatty acids (Figure 7). The amount of fatty acids increased to 79. 6 ± 1.9 μg/mg in Se:era versus 73.6 ± 2.7 μg/mg DW in PCC7942 (an 8% increase). This increase in fatty acids is mostly due to a significant rise in the amount of C16:0 (7%), C18:1 (34%) and to a far lesser extend C17:1 (26%). Together they account for 68% of the increase in total fatty acids, 42%, 26% and 1% for C16:0, C18:1 and C17:1 respectively.

No differences were observed in the amount of hydrocarbons compared to PCC 7942 (Figure 8).

### Fatty acid and hydrocarbon profile Se: Δera

Strain Se:Δera, containing the inactivated transposon, contains a similar amount (75.2 ± 1.0 μg/mg) of total fatty acids as PCC 7942 (73.6 ± 2.7 μg/mg DW) (Figure 7). Nevertheless, there are some significant changes in the amount of individual fatty acids. This strain has an 162% increase in the amount of C14:0 and an 93% increase in C14:1 compared to PCC7942. Furthermore, it has a 39% reduction in the amount of C18:0 and a 21% reduction in the amount of C18:1 versus PCC 7942.

This strain is the only strain with significant changes in the hydrocarbon profile (Figure 8). It contains a higher amount of pentadecane and 8-heptadecene compared to PCC 7942, 25% and 76% respectively. Furthermore, it has a substantial reduction (50%) in the amount of heptadecane.

### Fatty acid and hydrocarbon profile Se: Δera + era

Strain Se:Δera + era, containing the inactivated transposon complemented ectopically by era, has an almost identical amount of total fatty acids (73.8 ± 1.2 μg/mg) compared to PCC 7942 (73.6 ± 2.7 μg/mg DW) (Figure 7). The only fatty acid, which showed a significant difference, is myristic acid (C14:0). This fatty acid was increased by 31% compared to PCC 7942.

The hydrocarbons were similar compared to PCC 7942 (Figure 8).

## Discussion

### The *era* gene is essential for *S. elongatus* PCC 7942

In this study we generated a transposon library of *S. elongatus* PCC 7942 in order to isolate mutants with beneficial biodiesel production traits, including enhanced fatty acid and hydrocarbon content. The transposon mutant 2A01 was selected based on (i) increased staining with the lipophilic dye Nile red (indicating a higher lipid content), (ii) elongated cell morphology (might facilitate harvesting) and (iii) phenotypic stability. The transposon of mutant 2A01 was inserted into the 3 prime region of the Synpcc7942_0160 (GTP-binding protein Era) gene. Due to this insertion, a premature stop codon was introduced into the KH-domain of this protein. The KH domain is involved in RNA binding in *E. coli* and the GTP binding domain regulates its activity [16]. Moreover, all GTP-binding protein Era homologs that have been studied to date for membrane binding activity (*E.coli, S. pneumonia and S. mutans*) are associated with the cytoplasmic membrane [17]. This activity is most likely also linked with the KH domain which is well conserved in all GTP-binding protein Era homologs including that of *S. elongatus* PCC 7942 [18].

The *era* gene is the first gene of an operon containing a total of 8 genes. These include 5 genes encoding hypothetical proteins (most likely involved in nutrient stress, ribosome assembly and photosynthesis) and 3 that encode a tRNA-Met, an Iojap-like protein and the GTP-binding protein Era itself (Additional file 2: Table S3). Genes, which are grouped into operons together in multiple species, likely have similar functions even if transcribed individually in other species [19]. This suggests that *era* has a similar function in cyanobacteria as in *E. coli* in linking nutrient stress, to other cellular processes such as protein synthesis (ribosome assembly), photosynthesis and cell division [16].

To study whether the phenotype of the transposon insertion is (also) caused by a possible effect on the expression of downstream genes, we attempted to create a knockout of the *era* gene. We prevented transcription of all downstream genes by replacing the *era* gene with an antibiotic cassette flanked by two transcriptional terminators. Unfortunately, several transformation experiments with this cassette did not result in the isolation of the expected mutant, suggesting that one or several of the genes downstream of *era* are essential. However, subsequent introduction of a knockout cassette lacking the transcriptional terminators resulted in the isolation of a transient knockout mutant of *era*. After subcultivation of this knockout mutant, cells either failed to grow or the wild type phenotype was restored. Restoration of the wild type phenotype was associated with the removal of the resistance cassette from the *era* gene. These results indicates that *era* might also be an essential gene in PCC 7942 as was previously shown for *E. coli* [20]. Moreover, we found that the transient mutant also showed elongated cells and problems in septa formation. In addition, the deletion mutation was fully complemented by the introduction of an ectopic *era* gene (Additional file 1: Figure S4). We further verified the phenotype-genotype relationship by a) overexpressing era b) recreating the original transposon mutant, and c) complementing the recreated transposon mutant with an ectopic copy of era.

### GTP-binding protein Era is involved in lipid and hydrocarbon production of PCC 7942

Analysis of an *era* overexpression strain, Se:era, showed a significantly higher amount of fatty acids compared to PCC 7942 (Figure 7). These results demonstrate the importance of the GTP-binding protein Era for the production of fatty acids in *S. elongatus* PCC 7942.

To further verify the role of Era in biodiesel precursor production, we reintroduced the inactive transposon in PCC 7942 and complemented the resulted Se:∆era mutant with an ectopic copy of era. Strain Se:∆era does not show a significant difference in the total amount of fatty acids, compared to PCC7942 (Figure 7). However, because this strain has significantly longer cells (Figure 5), there are fewer cells per mg of dry weight compared to PCC 7942. These results indicate that there is a significantly higher amount of lipids per cell, supporting the original Nile red results. Moreover, several individual fatty acids are significantly different from PCC 7942 (Figure 7), suggesting that era plays a role in fatty acid synthesis in this strain. Complementation of Se:∆era with the ectopically integrated *era* gene (se:∆era + era) fully restored the fatty acid profile to PCC 7942 (except for C14:0), supporting the previous observation that Era is responsible for the changes in fatty acid profile.

In Se:era we did not observe any significant changes in hydrocarbon production. However, we found significant changes in hydrocarbon production in Se:∆era, which were restored when complemented with era (Figure 8), suggesting that Era is also involved in hydrocarbon metabolism.

Rational strain improvement for biodiesel precursor production of strain PCC 7942 has been attempted in the past by the introduction of thioesterase, acetyl-coA carboxylase and ribulose-1,5-bisphosphate carboxylase/oxygenase. However, these modifications failed to increase the net FA production [21]. Moreover, identified targets using RNA-seq to enhance the strain productivity of PCC 7942 led only to minor improvements in FA production [3]. However, our results show a substantial increase in fatty acid production without causing negative effects on biomass accumulation, suggesting that the GTP-binding protein Era is a new promising gene target for further strain improvement for the production of fatty acids (and possibly hydrocarbons).

### GTP-binding protein Era is important for cell size of PCC 7942

Several studies in *E. coli* showed that overexpression of *era* up to 5% of total cellular protein does not alter cellular growth, cAMP levels or protein production [22,23]. This is possibly due to the fact that the activity of the GTP-binding protein Era is regulated by binding of GTP [24]. However, we observed a significantly effect on cellular size during late exponential growth of PCC 7942 containing an additional *era* gene. Although the effect on cell size is subtle and can easily be missed during microscopic examination, cells of Se:era are significantly smaller than those of PCC 7942 (>11%; Figure 5). To our knowledge, this is the first report demonstrating that overexpression of Era causes a change in cell size.

Depletion of Era in *E. coli* causes a decrease in growth, loss of viability, septum formation and elongated cells [17]. In the mutant se:∆era, containing the inactivated transposon, we did observe an effect on septum formation and cell size. However, this mutant did not show a difference in growth rate nor viability compared to PCC 7942. Analysis of the phenotype of se:∆era suggests that the expression of *era* might be reduced to a level which is slightly lower than that required for normal cell division [23].

Introduction of an ectopic era in strain se:∆era resulted in restoring the formation of septa in the middle of a cell, but not entirely the cell length. While more than 96% of all the cells were within the same length range as PCC 7942, some cells were up to 26% larger than WT. However, the median length of these cells was 31% smaller compared to strain Se:∆era. These results suggest the insertion in era itself and/or one or more downstream genes is responsible for the elongated cell morphology.

The fact that introduction of an additional *era* gene did not fully complement the Se:∆era mutant, might have several reasons. First of all, the expression of the era gene used for complementation is not regulated by its native promoter. For example, it has been shown that expression of *era* is coupled to cellular growth (fast growth > Era) in *E. coli* [23]. Another possibility is that downstream genes might be responsible for part of the phenotype and/or presence of a truncated era interferes with the complementation.

## Conclusions

It was previously shown that Era plays a role in many cellular processes including ribosome assembly and cell cycle regulation [17]. This protein has been well studied in diverse bacterial species, but never in cyanobacteria. In this report we showed that *era* is an essential gene for *S. elongatus* PCC 7942. In this strain, the GTP-binding protein Era is involved in hydrocarbon and fatty acid metabolism as well as the determination of cell size. A possible explanation for the observed changes in fatty acid and hydrocarbons is the role that Era plays in coupling nutrient stress, cell division and photosynthesis. In a normal situation Era delays cell division and synthesis of fatty acids, causing cells to become longer and synthesize less proteins and fatty acids. In the Era overexpression strain (Se:era), this regulation is slightly disrupted causing almost no change in cellular size (3.3 μm versus 3.4 μm in the stationary phase) and a relative increased fatty acid synthesis.

We propose that the Era protein can be used as a novel target for improving biofuel production traits. It would be interesting to evaluate the effect of overexpression of *era* in combination with other genetic modifications, for example in a strain expressing a thioesterase and containing a knockout of the acyl-ACP synthetase. Furthermore, the question remains open whether overproduction of the GTP-binding protein Era also influences cellular size and fatty acid/hydrocarbon content in other bacteria.

## Methods

### Bacterial strains and plasmids

Wild-type and mutant *E. coli* and *S. elongatus* PCC 7942 strains are shown in Table 1. Table 1 further lists the plasmids that were used in this study. Primers used for vector construction and verification are listed in Table 2.

**Table 1:** **Strains and plasmids used in this study**

	**Description**	**Reference**
**Strain**		
*E. coli* DH5α	Used for molecular cloning	[25]
*S. elongatus* PCC 7942	Wild-type freshwater cyanobacterium	gifted by Susan S. Golden (UCSD, California)
2A01	*S. elongatus* PCC 7942 containing transposon pRL1063a	This study
PCC 7942	*S. elongatus* PCC 7942 containing pNS3	This study
Se:era	*S. elongatus* PCC 7942 expressing the *era* gene from neutral site 3	This study
Se:Δera	*S. elongatus* PCC 7942 with a insertion of the inactivate transposon (pSE3) and expressing the *era* gene from neutral site 3	This study
Se:Δera + era	*S. elongatus* PCC 7942 expressing a truncated thioesterase from *E. coli* DH5α (*'tesA*) and containing a disrupted acyl-ACP synthetase (SynPCC 7942_0918)	This study
**Plasmid**		
pJet1.2	Used for the cloning of blunt PCR products	Thermo-Fisher Scientific (Waltham, MA, USA)
pRL1063a	Contains a transposon based on Tn5, which bears several antibiotic resistance genes (kanamycin (Km), bleomycin (Ble) and streptomycin (Sm)), promoterless luciferase (*luxAB*) reporter genes, an oriV not recognized by PCC 7942 and a transposase gene. The oriT for conjugative transfer is present on the plasmid but is not part of the transposon.	[11]
pHN1-LacUV5	Targets Neutral site 3, confers resistance to chloramphenicol antibiotic (Cm) and contains a strong isopropyl-β-D-thiogalactopyranoside (IPTG)-regulated lacUV5 promoter followed by a ribosome binding site (RBS) and an unique HindIII restriction site	[15]
pNS3	Derived from pHN1-LacUV5 with the original RBS removed and lacking the ATG start codon near the multiple cloning site	This study
pNS3:ERA	Derived from pNS3 with *era* gene (Synpcc7942_0160) cloned into the unique HindIII restriction site behind the inducible lacUV5 promoter	This study
pSe:Δera	Inactivate transposon TN5-1063a originally isolated from strain 2A01, with the transposase mutate by digestion with NotI and blunting using T4 DNA polymerase	This study

**Table 2:** **Primers used in this study**

**Primer**	**Sequence** ^**a**^	**Remarks**
5pERAfw	ctcgaggtaggggttgatctcgtgga	Used in combination with pRL1063a_rev and ERA3pRev2 for segregation check
ERA3pRev2	ggcaaacgctgaaagtcttc	Amplifies the 3 prime region outside the inactive transposon construct pSe:∆era. Used to check the segregation in combination with pRL1063a_fw and 5pERAfw
ERAfw	*aagctt* **aaggaggaaaaa**atgtccgaccttttcaccac	Primer for amplifying the *era* gene, addition of HindIII restriction site and ribosome binding site
ERArv	*aagctt*ttactcactctcaggtcggtagc	Primer for amplifying the *era* gene, addition of HindIII restriction site
pRL1063a_fw	aggaggtcacatggaatatcagat	Used for sequencing flanking regions of TN5-1063a
pRL1063a_rev	tactagattcaatgctatcaatgag	Used for sequencing flanking regions of TN5-1063a

^a^Restriction sites are indicated in italics and the ribosomal binding site is indicated in bold.

### Culture conditions

PCC 7942 and its mutants were cultured in BG-11 medium at 30°C. Liquid cultures were incubated on a rotary shaker at 250 rpm under continuous light (60 μE/m^2^/s). When needed antibiotics were added to a final concentration of 25 μg/ml kanamycin and/or 10 μg/ml chloramphenicol. Cellular growth was routinely determined by taking an aliquot of 1 ml every 24 hours for 5 days, diluted to an appropriate optical density and the absorption was determined at 750 nm.

### Transposon library construction

To perform transposon mutagenesis, plasmid pRL1063a was used [11]. Plasmid pRL1063a contains a transposon based on Tn5, which bears several antibiotic resistance genes (kanamycin (Km), bleomycin (Ble) and streptomycin (Sm)), promoterless luciferase (*luxAB*) reporter genes, an oriV not recognized by PCC 7942 and a transposase gene. The oriT for conjugative transfer is present on the plasmid but is not part of the transposon. Conjugation was used to introduce pRL1063a into PCC 7942, using a method similar to that of Clerico et al. 2007 [26]. After 3 days of growth under low light conditions (5 μE/m^2^/s), kanamycin was added underneath the plates to a final concentration of 25 μg/ml and plates were re-incubated at 30°C under normal light conditions (60 μE/m^2^/s) until colonies formed.

### Screening for increased lipids using flow cytometry and nile red

To get a qualitative indication of lipid content, mutant cells were stained using the lipophilic dye Nile Red [13]. Nile Red is a dye that becomes strongly fluorescent when present in a hydrophobic environment (e.g. membrane lipids). Depending on the hydrophobicity of the compounds, the dye emits either a yellow or red fluorescence. The intensity of the signal is an indication for the amount of lipids present. An aliquot of cell (1ml) from an exponentially growing culture was taken, transferred to an eppendorf tube and Nile red (1 mg/ml in DMSO) was added to a final concentration of 1 μg/ml. Cells were examined using a guave easyCyte flow cytometer (Merck), by excited with a 485nm laser and determining several parameters such as cellular size (forward scatter), complexity (side scatter) and yellow fluorescence (583/26 nm).

### Determination of transposon insertion site

To determine the site of transposition, cyanobacterial genomic DNA from mutant 2A01 was extracted using the method of Clerico *et al.* [26]. Subsequently, 10 μg of chromosomal DNA was digested overnight with EcoRI, re-ligated using T4 ligase and 5 μg DNA from this mixture was transformed into chemically competent *E. coli* DH5α cells. After plaiting the cells on LB medium containing 50 μg/ml kanamycin, colonies that formed after overnight cultivation at 37°C were inoculated into liquid LB medium and plasmid DNA was isolated and analysed by restriction analysis and sequencing.

### Construction of Era overexpression mutant and control

To elucidate the function of Era for *S. elongatus* PCC 7942, an overexpression vector (pNS3:ERA) was constructed. This vector was constructed using vector pNS3, which contains amongst others a chloramphenicol resistance marker, IPTG inducible promoter and homologous regions for integration at neutral site III. The *era* gene was amplified from PCC 7942 genomic DNA with primers ERAfw (containing a HindIII restriction site and ribosomal binding site) and ERArv (containing a HindIII restriction site) (Table 2). The resulting product was digested and ligated into a pNS3 vector, which resulted in the final plasmid pNS3:ERA. Transformation of this vector into PCC 7942 resulted in strain Se:era. At the same time, transformation of pNS3 into PCC 7942 was done to create a negative control.

### Construction of Era disruptions and complementation

To reconstruct the original phenotype caused by the transposon found in 2A01, the re-isolated transposon of 2A01 was inactivated and reintroduced into PCC 7942 as follows. The plasmid was digested using EcoRV to reduce the size of the vector (from app. 20 kb to 11 kb by removing part of the genomic DNA from this plasmid) and thereby making subsequent cloning steps easier. This smaller plasmid was linearized using NotI, which cuts in the transposase gene blunted using T4 DNA Polymerase and re-ligated. This vector (pSe:∆era) was transformed into strain Se:era, creating strain Se:∆era + era and PCC 7942 containing pNS3 creating strain Se:∆era.

### Analysis of cellular length

To see the effect of the introduced mutations on bacterial cell length, pictures were taken with a phase contrast microscope (Carl-Zeiss, Sliedrecht, The Netherlands) and analysed using ImageJ [27] with the Coli-Inspector plugin (Norbert Vischer, Bacterial Cell Biology, University of Amsterdam). This plugin is able to determine the cell length and diameter of individual cells in an automated manner. From this data a histogram was constructed and the median cell length was determined.

### Extraction of hydrocarbons and fatty acids for GC-MS

Extraction of hydrocarbons and fatty acids was performed as previously described in Voshol et al. 2014. Briefly, cells from a 5 day old culture were diluted to a start OD750 of approximately 0.05 and grown in BG-11 for 5 days as described above (in quadruplo). Bacterial cells were subsequently harvested by centrifugation (20 min, 4500 rcf), the supernatant was discarded and the pellet was freeze-dried. To each 20 mg of lyophilized biomass, 50 μl of internal standard (C10:0, 5 mg/ml), 1 ml Hexane and 2 ml methanol containing 0.5 M sodium methoxide was added. The samples were vortexed for 30 seconds and sonicated for 5 minutes. Subsequently they were incubated at 50°C for 10 minutes after which 3 ml 5% HCL in methanol was added to stop the reaction. The samples were then vortexed (30 seconds), sonicated (5 minutes) and incubated at 70°C (20 minutes). Samples were allowed to cool to room temperature and were extracted twice with 4 ml of hexane containing 50 mg/L butylated hydroxytoluene. The samples were then vacuum evaporation, dissolved in 1 ml of hexane and transferred to GC-MS vials.

### GC-MS analysis

These extracted hydrocarbons and fatty acids mixtures were analysed using an Agilent model 7890A gas chromatograph as previously described (Voshol et al. 2014). Briefly, samples were inserted into a model 7693 autosamples, separated using a DB-WAX column (10 m, 0.25 mm, 0.25 μm) and detected using an inert XL mass spectrometer (model 5975C) using helium as carrier gas. The initial oven temperature was 50°C for 1 minute, then the temperature was increased to 230°C (25°C/min until 200°C and 3°C/min until 230°C) after which this temperature was held for 8 minutes. Retention time and the mass spectrum of authentic samples were used for identification. Hydrocarbons were quantified relative to the internal standard while absolute quantities of fatty acids were determined based on calibration curves constructed using authentic standards.

### Statistics

R *Statistical* Software (Foundation for *Statistical* Computing, Vienna, Austria) was used to carry out the data analysis. Data were first tested using Leven’s test to indicate whether their variances were significantly different. Data that did not show a significant difference using Leven’s test were subsequently compared using ANOVA and post-hoc Tukey Honest Significant Differences test. Data that were not normally distributed or showed unequal variances were analysed using the nonparametric Mann–Whitney-Wilcoxon test. Differences were considered significant if their p values were smaller than 0.05.

## Additional files

Additional file 1:
**Additional figures.** Red **(Figure S1)** and Green **(Figure S2)** fluorescence versus front scatter as determined using flow cytometry. Domain organization of the GTP-binding protein Era of *S. elongatus* PCC 7942 **(Figure S3)** and phase-contrast microscopic view of *S. elongatus* PCC 7942 and *era* knockout mutant where the complete coding region of the *era* gene was replaced by a Km resistance cassette. Additional file 2:
**Additional tables.** Quantity of fatty acids **(Table S1)** and hydrocarbons **(Table S2)** of PCC 7942 and its mutants. **Table S3** contains the original annotation of the eight genes of the *era* operon and their putative function.
