# Supplementary material for: GTP-binding protein Era: a novel gene target for biofuel production
Source: BMC Biotechnol. 2015 Mar 24;15:21. doi: 10.1186/s12896-015-0132-1 (PMC4380250; doi:10.1186/s12896-015-0132-1)
Supplement: Additional file 1: — Additional figures. Red (Figure S1) and Green (Figure S2) fluorescence versus front scatter as determined using flow cytometry. Domain organization of the GTP-binding protein Era of S. elongatus PCC 7942 (Figure S3) and phase-contrast microscopic view of S. elongatus PCC 7942 and era knockout mutant where the complete coding region of the era gene was replaced by a Km resistance cassette. [file 12896_2015_132_MOESM1_ESM.pdf]

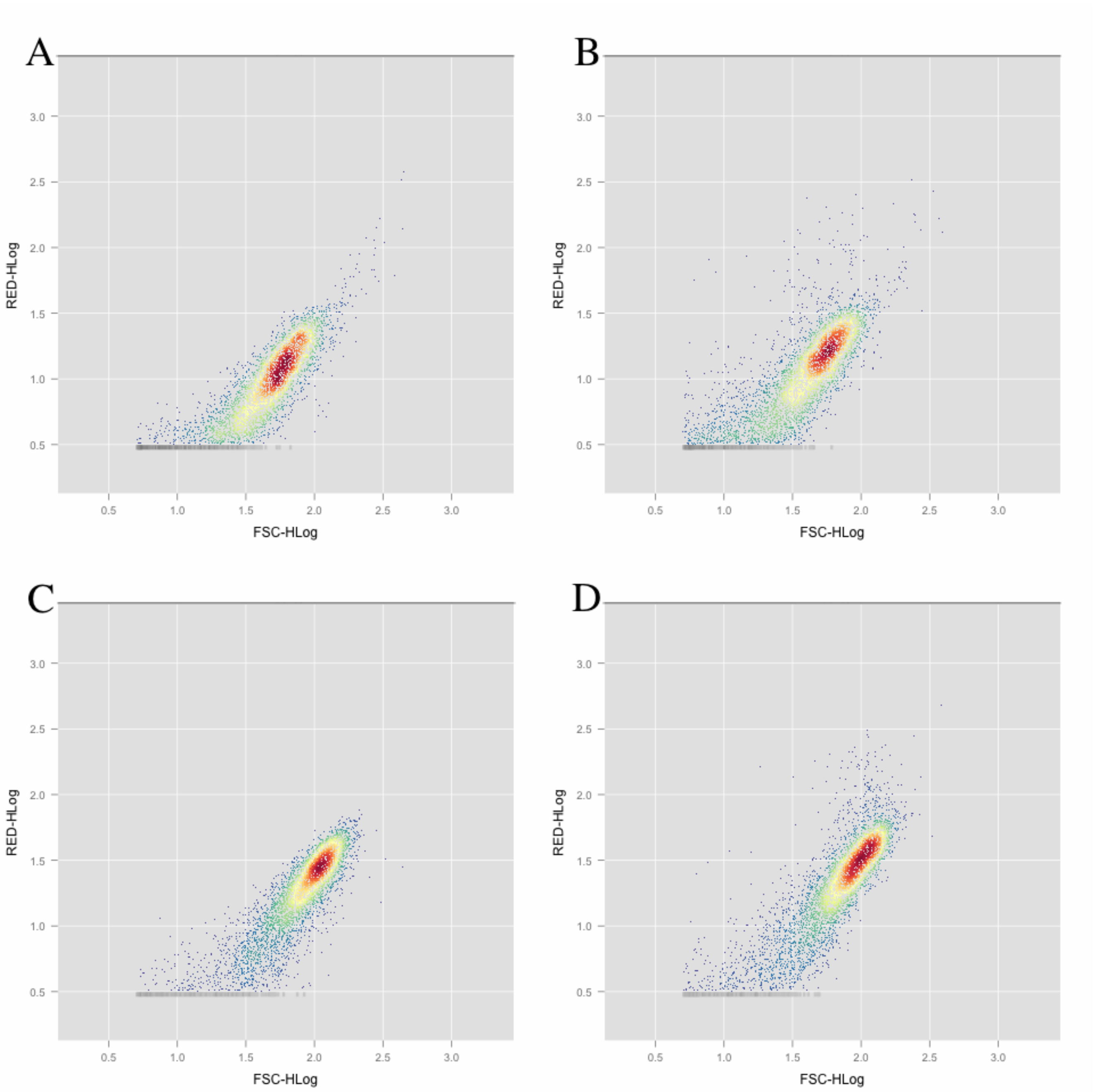

Figure S1 - Red fluorescence versus front scatter (cell length) as determined using flow cytometry of PCC 7942 (A and B) and mutant 2A01 (C and D) grown in liquid media for 48 hours either unstained (A and C) or stained (B and D) with the lipophilic dye Nile red

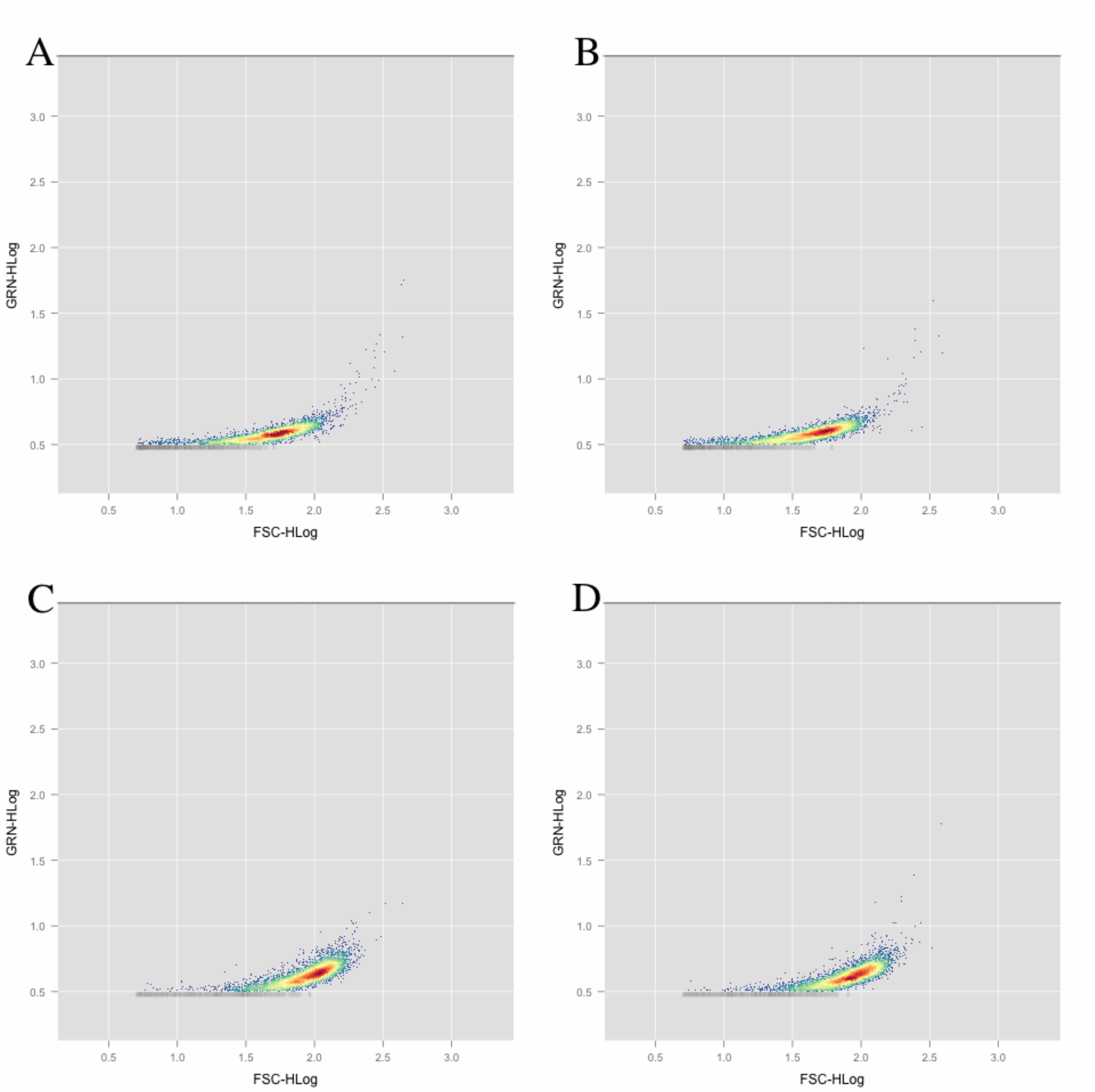

Figure S2 - Green fluorescence versus front scatter (cell length) as determined using flow cytometry of PCC 7942 (A and B) and mutant 2A01 (C and D) grown in liquid media for 48 hours either unstained (A and C) or stained (B and D) with the lipophilic dye Nile red

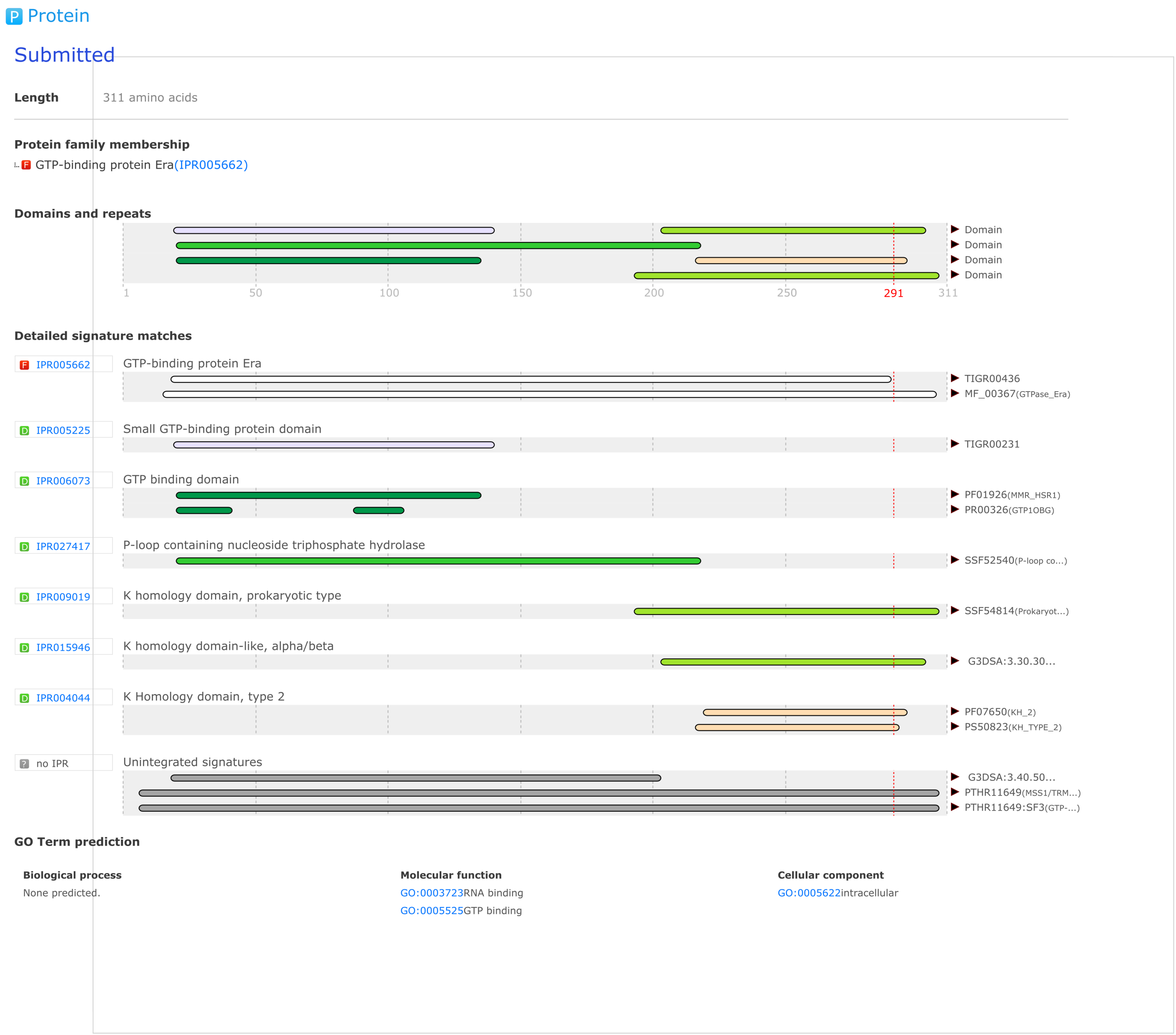

Figure S3 – Domain organization of the GTP-binding protein Era of *S. elongatus* PCC 7942. The Era protein was scanned against the InterPro collection of protein signature databases. The red dashed line indicates the location of the stop codon introduced by the transposon (291 amino acids).

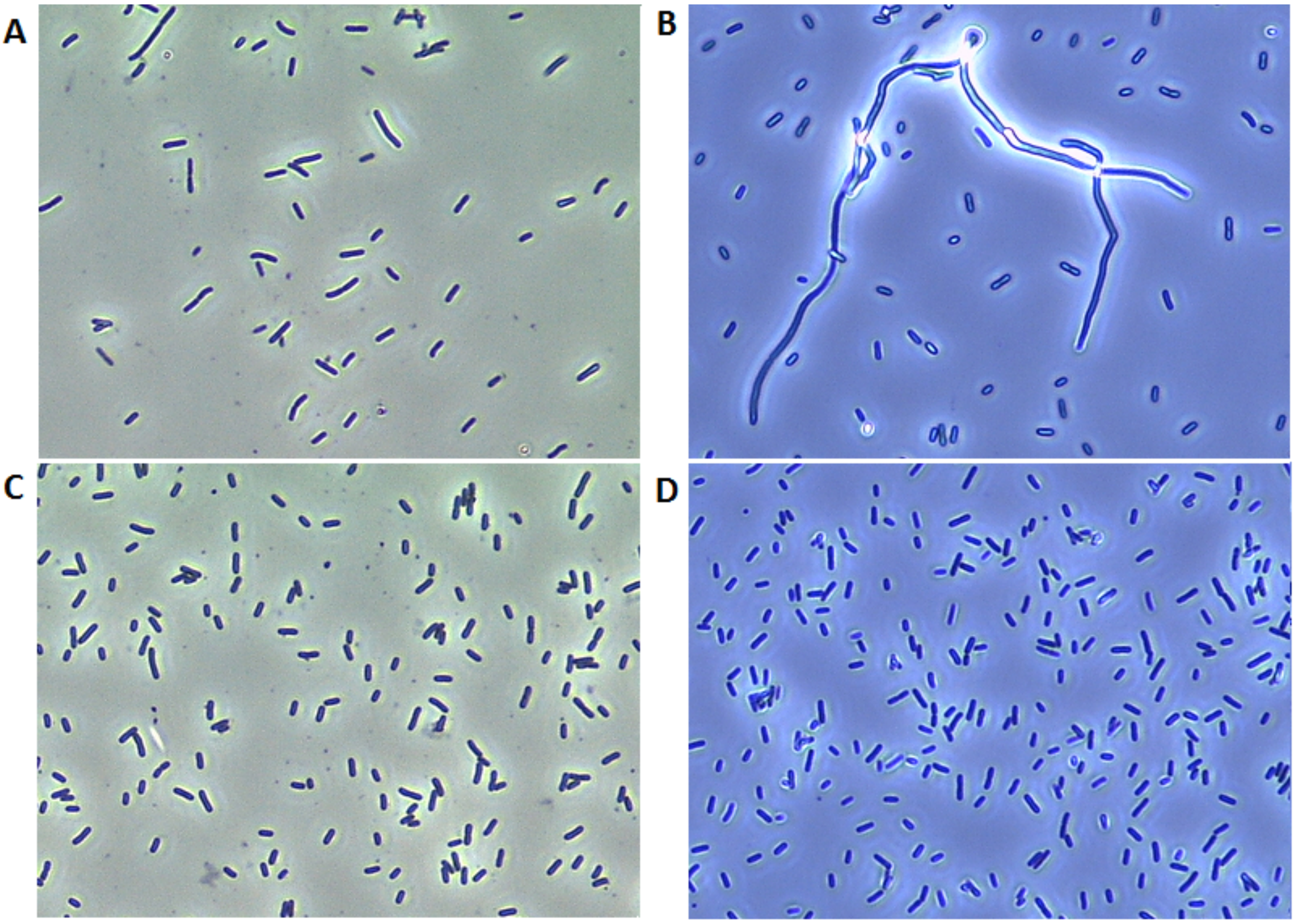

Figure S4 - Phase-contrast microscopic view of *S. elongatus* PCC7942 and its mutants (400x magnification). (A) Wild *S. elongatus* PCC 7942, (B) an *era* knockout mutant where the complete coding region of the *era* gene was replaced by a Km resistance cassette, (C) similar knockout mutant complemented with pNS3:ERA and (D) the knockout strain (shown in B) after three days of growth in liquid BG-11 medium.
